# Fourier transform spectroscopic identification of an intraocular foreign body

**DOI:** 10.1016/j.ajoc.2022.101778

**Published:** 2023-01-02

**Authors:** Anh D. Bui, Jack Dickson, Carol Hoerner, Ricardo Lamy, Jay M. Stewart

**Affiliations:** a University of California, San Francisco, Department of Ophthalmology, San Francisco, CA, United States; b Zuckerberg San Francisco General Hospital and Trauma Center, Department of Ophthalmology, San Francisco, CA, United States

A 74-year-old woman reported an unusual floater three days after undergoing a pars plana vitrectomy and lens exchange with scleral fixation for a dislocated intraocular lens. Fundus examination revealed a dark fiber-like foreign body in the vitreous cavity with no associated intraocular inflammation [Fig. 1A**,** magnified (**inset**)]. The fiber was removed and measured 15.4 mm in length and 61 μm in diameter (Fig. 1B) and appeared similar to a surgical huck cotton towel fiber (Fig. 1C). Spectral images of the foreign body and a towel fiber were captured by Fourier transform infrared spectroscopy (FTIR) and background corrected. The absorbance spectra of both specimens matched (Fig. 1D), suggesting that the foreign body originated from the surgical towel. The patient reported subjective improvement in symptoms after removal of the fiber. This case demonstrates the applicability of FTIR for identification of intraocular foreign bodies and the importance of inspecting instruments which had been resting on surgical towels.

**Fig. 1:**
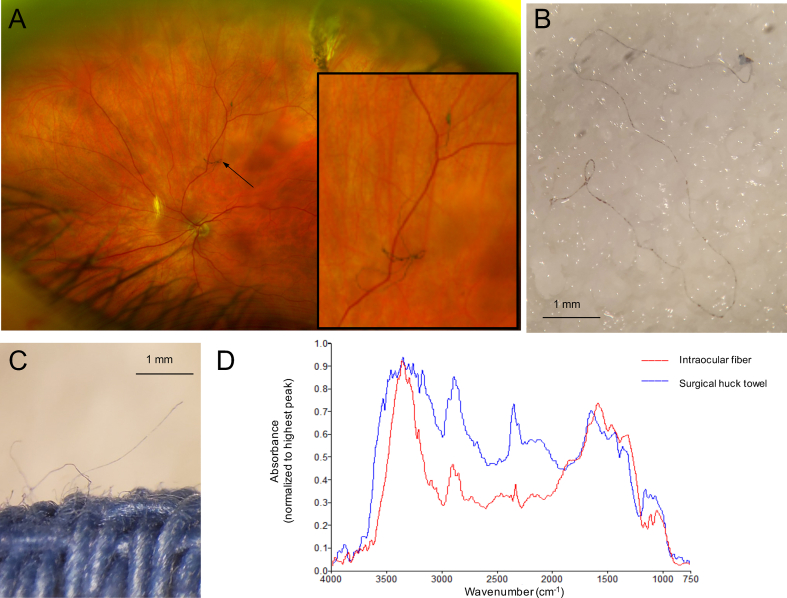
Fundus photograph revealing the intraocular fiber [A, magnified (inset)]. Bright field microscopy image of the removed intraocular fiber (B) and of a surgical huck towel (C). Normalized absorbance spectra of the removed intraocular foreign fiber and a surgical huck towel fiber obtained by Fourier transform infrared spectroscopy (D).

## Funding

Research to Prevent Blindness and All May See Foundation.

## Declaration of competing interest

None reported.

